# Routine Sensitive Enquiry of Adult Interpersonal Trauma in Community Mental Health Teams: An Audit of the Initial Assessment Tool

**DOI:** 10.1007/s10597-023-01220-z

**Published:** 2024-02-16

**Authors:** Natasha Hill, Nicholas Graham, Rebecca L. Forrester

**Affiliations:** 1 Department of Clinical Psychology, Gartnavel Hospital, University of Glasgow, Glasgow, Scotland, UK; 2 Dykebar Hospital, NHS Greater Glasgow & Clyde, Grahamston Road, Glasgow, Scotland, UK; 3 Arndale Resource Centre, NHS Greater Glasgow & Clyde, Kinfauns Road, Glasgow, Scotland, UK

**Keywords:** Assessment, Trauma, Enquiry, Abuse, Mental Health

## Abstract

This audit aimed to evaluate the utility of the Initial Assessment Tool (IAT) in documenting routine sensitive enquiry of adult interpersonal trauma within three Community Mental Health Teams (CMHTs) in North-East Glasgow. In addition, it sought to evaluate if disclosures informed patient risk assessments and if patients were signposted to additional support services. 57% of 90 IATs had evidence of routine sensitive enquiry. Of 51 casefiles with evidence of routine sensitive enquiry, 61% had evidence of the information informing their risk assessments and 14% had documented recommendations of support organisations. The IAT appeared able to assist clinicians with routine sensitive enquiry in adulthood. However, there may be advantage in supporting staff understanding of how to ask questions to specific populations and to use this information to inform treatment planning. Given the prevalence of adult interpersonal trauma experienced by patients presenting to CMHTs, trauma-informed approaches to care should be implemented.

**Supplementary Information:**

The online version contains supplementary material available at 10.1007/s10597-023-01220-z.

## Introduction

Across the UK, interpersonal trauma—including physical, emotional and sexual abuse—amongst adult populations remains a public health and human rights issue (Chandan et al., 2020; Pedersen et al., 2021). For example, in Scotland, recent reports indicate 20% of women and 4% of men have experienced sexual assault in adulthood (Public Health Scotland, 2021). The prevalence of adult interpersonal trauma is replicated in a recent report from the Crime Survey for England and Wales, which make an annual estimation of 2.4 million adults having experienced domestic abuse (Elkin, 2022). In its most severe form, interpersonal trauma can result in death, with particular risk for men indicated as 70% of all homicide victims between 2021 and 2022 in Scotland were male (The Scottish Government, 2022). By contrast, when considered through the lens of intimate partner violence, women have been found to be disproportionally affected as 52% of women murdered between 2012 and 2022 were killed by a partner or ex-partner, compared with 4% of men murdered in the same ten-year period (The Scottish Government, 2022). Beyond the risks of physical harm and death resulting from interpersonal trauma in adulthood, there are strong associations between such traumas and long-term poorer mental health and wellbeing (MacMillan et al., 2001). This includes higher incidences of posttraumatic stress disorder, anxiety, depression and suicide amongst adult abuse victims (World Health Organisation, 2013). Adults accessing mental health services are significantly more likely to have experienced some form of abuse (Mauritz et al., 2013), and to have experienced domestic or sexual violence in the previous year (Khalifeh, et al., 2015), compared with the general population. This appears to have an interactive effect as having a mental health problem increases your risk of being a victim of abuse (Christ et al., 2020), as well as increasing the risks of revictimization (Classen et al., 2005).

Policies across the UK are increasingly recognising the long-lasting impacts of the broad range of abuse including physical, emotional, sexual and financial abuses on adult mental health (Bellis et al., 2014). Scottish policy, policing and legislation are also making developments in recognising the impact of domestic abuse and its roots in structural gender inequality through initiatives such as Equally Safe: Scotland’s Strategy for Preventing and Eradicating Violence against Women and Girls report (The Scottish Government, 2014). Despite this, some reviews have concluded that abuse can often be misunderstood by health care practitioners (Watson, 2017) and a recent Whole Lives Scotland report in 2017 identified a lack of consistency in the response to abuse across Scotland (Safe Lives, 2017). These inconsistencies have been coined a “postcode lottery” for service users, survivors and families. These inconsistencies have been reflected in a recent review of Community Mental Health Services in England which indicated only 13% of patients’ clinical records documented evidence of adverse experiences (Neill & Read, 2022).

Trauma-informed practice intends to provide a prominent role to the pervasive impact of trauma on a person’s relationships and ways of interacting with the world (The Scottish Government, 2021a). Services that endorse a trauma-informed approach have growing evidence of effectiveness in fostering hope and empowerment for victims of abuse, as well as assisting clinicians working with this population (Mead & Filson, 2017). Consequently, there has been a drive for all health services, including CMHTs, to move towards trauma-informed care and practice (Moloney et al., 2018). One way in which CMHTs in Scotland are attempting to use a trauma-informed approach and improve early identification of those who have experienced—or may still be experiencing—abuse is through routine sensitive enquiry (Young et al., 2019). This involves asking all new patients at assessment about experiences of abuse, regardless of whether there are any indicators or suspicions of abuse. This term has been used within other healthcare settings, for example, postnatal care (Boddy, 2020). With improvements to early identification, the aim is that there will be parallel improvements seen in support and treatment offered to service users at possible risk of harm.

CMHTs across NHS Greater Glasgow & Clyde (GG&C) have attempted to standardise care and create consistent practices of assessment with the introduction of the Initial Assessment Tool (IAT) (Online Appendix 1). The IAT is a comprehensive mental health assessment and includes questions relating to routine sensitive enquiry. It was designed through the Effective and Efficient Community Mental Health Services Steering Group in 2018 to replace existing specialist assessments and related assessment tools being used across the Health board. The IAT Principles and Guidance document (Online Appendix 2) states that the IAT should be used for all new routine referrals within Adult CHMTs. There is also a Brief IAT (BAT) which can be used for urgent and emergency assessments and if a full assessment has been completed within the previous 6 months. A full IAT is required to be completed within 28 days of a patient’s initial contact, if they continue to receive input from the service. Part of the IAT is used to assist staff to enquire about interpersonal trauma in adulthood with the aid of specific questions related to routine sensitive enquiry. Clinicians use the IAT via an existing template on EMIS Web, the electronic notes system used in NHS GG&C, which form part of patients’ medical record. In doing so, it is hoped that a shared protocol would increase early identification of historical and current abuse, improve risk assessment and management plans and appropriately target interventions for service users (Trevillion et al., 2016). This may be especially pertinent given evidence that social isolation resulting from the COVID-19 pandemic has increased risk for victims, making help-seeking more difficult (Bradbury-Jones & Isham, 2020).

## Aims

This audit aimed to evaluate the utility of the Initial IAT in documenting routine sensitive enquiry of interpersonal trauma in adulthood in three Community Mental Health Teams (CMHTs) in North-East Glasgow. The comparator guidance used as a standard was the Initial Assessment Tool General Guidance document (Online Appendix 1), specifically whether the questions relating to routine sensitive enquiry of adult interpersonal trauma had been documented. As this is the first audit of routine sensitive enquiry using the IAT, we aimed to achieve a baseline as part of an audit cycle.

It set out to answer three questions:

1. Is routine sensitive enquiry of adult trauma being reported within the IAT during new patient assessments?
2. For patients who disclosed adult interpersonal trauma, is the information gathered informing their risk assessments?
3. For patients who disclosed adult interpersonal trauma**,** are recommendations of support through relevant organisations (in addition to, or instead of CMHT interventions) being documented within the IAT?

The scope of this audit was limited to focussing on adult interpersonal trauma; rather than an audit of interpersonal traumas experienced across the lifespan. This was decided as the IAT’s questions on adult interpersonal trauma disclosures are distinct from questions related to traumas experienced in childhood. By increasing the specificity of the audit question, the authors believed there would be improved understanding on the current utility of this section of the IAT. In doing so, authors hoped to improve the learning points and recommendations for CMHTs.

## Methods

### Research Design

This project was a retrospective audit whereby the casefiles of 90 new patients, across three CMHTs in North-East Glasgow, were reviewed to examine whether questions relating to routine sensitive enquiry of adult interpersonal trauma were reported during initial assessment using the IAT. These questions include enquiry into experiences of physical, emotional, sexual or financial adult abuse and experiences of intimate partner violence and additional questions are outlined to ask to women:

- Have you ever experienced physical, psychological or sexual abuse or violence within any of your intimate relationships?
- Does the violence you’ve experienced still affect your well-being, health or life?
- Is there any physical, psychological or sexual violence or abuse in your current intimate relationships?

### Participants

Data for this audit was collected from the casefiles of 90 patients across three North East CMHTs. Specifically, the researcher accessed the 30 most recent assessments from each North-East CMHT that had a completed IAT on their EMIS records. To stratify data collection, the researcher took 10 patients from the nursing ‘treatment waiting’ caseloads, 10 from the psychology ‘treatment waiting’ caseloads and 10 from the occupational therapy ‘treatment waiting’ caseloads for each team and screened to ensure there were no duplications. The final sample reflected a variety of clinicians completing the initial assessment and included all qualified CMHT disciplines (Nursing, Medical, Occupational Therapy and Psychology).

In terms of inclusion criteria, a patient was considered eligible if they had a completed IAT uploaded onto their electronic EMIS records. The researcher reviewed a total of 140 casefiles before reaching the full sample of 90 patients, meaning there were 50 patients who were excluded due to only having a BAT or no IAT documentation on their EMIS records.

All genders were included in this audit as there was recognition of the incidence of and risks to mental health for female (Pemberton & Loeb, 2020), male (Huntley et al., 2019) and transgender people (Peitzmeier et al., 2020).

### Procedure

Once the researcher had reached 90 patients with completed IAT uploaded to their EMIS records, they began the process of examining whether there was evidence of routine sensitive enquiry of adult interpersonal trauma, as per guidance set out in the IAT General Guidance Document (Supplementary Information 1). A ‘completed routine enquiry’ was operationalised as the patient’s IAT having clear documentation that the questions relating to routine sensitive enquiry of adult interpersonal trauma had been reported. For example, if the section was left blank, the researcher would note this as ‘incomplete’ as they could not assume that the questions had been asked. If the clinician completing the assessment has documented ‘nil abuse’ or similar, this was considered a completed enquiry.

To answer the second audit question, the researcher looked at whether information gathered from routine sensitive enquiry informed what was documented on the Clinical Risk Assessment Framework for Teams (CRAFT). The CRAFT is an electronic template found on EMIS that is completed following every initial assessment appointment. On the CRAFT template, there is a section specifically for ‘risk from others’. A patient was considered to have ‘evidence of informed CRAFT’ if their risk assessment documented information relating to adult interpersonal trauma in the ‘risk from others’ section. For example, if someone had disclosed experiencing domestic violence as an adult in the IAT, this would be specifically noted in the ‘risk from others’ section.

Finally, the researcher evaluated whether patients whose files had evidence of routine sensitive enquiry using the IAT were documented as being offered any further support following their initial assessment. The operationalisation of ‘further support’ included any documentation of signposting or additional advice to engage with third sector organisations or services outside of the CMHT, for example, Women’s Aid. To do this, the researcher looked for documentation in the patients’ CRAFT risk assessment, IAT tool and clinical assessment notes, for evidence this was discussed, or offered.

## Results

Once EMIS data was collected for the 90 patients, across the 3 CMHTs, it was possible to summarise the characteristics of the sample. Patients were seeking assistance from the CMHT for a variety of mental health difficulties. Table 2 shows the broad range of referral reasons noted in patients’ IATs and number of patients seeking support for that presenting problem. Depression was the most cited reason for referral (28.9% of overall sample).

For the 90 patients’ casefiles used in this audit, 51 were White Scottish (57%) and 9 were White British (10%) which was considered reflective of the local population in North-East Glasgow (See Table 1).

**Table 1:** Characteristics of the sample of 90 casefiles audited

		*N*
Ethnicity	White Scottish	51
	White British	9
	White Other	2
	African	1
	Arabic	3
	Black Caribbean	1
	Mixed	1
	Other Ethnic Group	3
	Unknown	19
	Total	90
Age Range	18–25	21
	26–30	5
	31–40	28
	41–50	17
	51–60	9
	Total	90
Gender	Male	32
	Female	58
	Total	90
Main Diagnoses	Mood disorders Anxiety disorders Eating disorders Psychotic disorders Substance use disorders Personality disorders Post-traumatic Stress Disorders Total	35 7 3 17 3 11 14 90

There was a range of ages represented in the total sample with 31% (n = 28) of patients being between ages 31–40. Therefore, the sample was likely to include people exposed to interpersonal trauma as recent figures indicate that 31–35-year-olds had the highest incidence rates for both victims and perpetrators of abuse (The Scottish Government, 2021b).

Patient gender was evaluated in this audit with 64% of the total sample identifying as female (n = 58) and 36% identifying as male (n = 32). Of the staff completing initial assessments, 81% were nursing staff (n = 73). Assessments were also completed by crisis practitioners (n = 5), doctors (n = 5), occupational therapists (n = 5) and clinical psychologists (n = 2).

Audit question 1: Are questions relating to routine sensitive enquiry of adult interpersonal trauma being reported within the initial assessment tool during new patient assessments? (n = 90).

Of the 90 patients evaluated in this audit, 51 (57%) had evidence that questions pertaining to routine sensitive enquiry of adult interpersonal trauma were asked and documented using the IAT. For the remaining 39 patients (43%), there was no documentation of the questions relating to routine sensitive enquiry being asked within their IAT. Of the 51 patients with evidence of routine sensitive enquiry, 42 (82%) were female and 9 (18%) were male. There was a significant positive correlation between gender of participant and evidence of routine sensitive enquiry r_s_ (88) = 0.43, p < 0.01. There was no significant correlation found between psychiatric diagnoses and having evidence of routine sensitive enquiry r_s_ (88) = -0.12, p = 0.244.

Audit question 2: For patients who disclosed adult interpersonal trauma, is the information gathered informing what is documented in their CRAFT risk assessments? (n = 51).

Audit questions 2 and 3 relate to patients who disclosed adult interpersonal trauma using the IAT and, as such, results relate to 51 out of 90 in the total patient sample.

To answer question 2, the researcher evaluated how many casefiles with documented routine sensitive enquiry in their IAT (n = 51) had evidence that this information informed their CRAFT risk assessment. A CRAFT risk assessment was operationalised as “informed” if there was evidence that information disclosed from routine sensitive enquiry within the IAT was also documented in the “risk from others” section of the CRAFT template. For example, if someone had experience of domestic violence, this would be reflected within the “risk from others” section of the CRAFT. If relevant information was missing, or no CRAFT was uploaded, these patients were classed as not having an “informed CRAFT”. 32 of the 51 patients (63%) had evidence of the information they disclosed through routine sensitive enquiry within the IAT also being documented within their CRAFT risk assessment completed following the assessment appointment.

Audit question 3: For patients who disclosed adult interpersonal trauma***,*** are recommendations of relevant support organisations being documented within the IAT? (n = 51).

The final audit question looked at whether routine sensitive enquiry using the IAT led to the offer of additional support for service users in the 51 patients who disclosed interpersonal trauma. The operationalisation of ‘further support’ was kept relatively flexible and included any signposting or referral to relevant services or support organisations beyond, or in addition to, CMHT treatment, such as Women’s Aid. In this evaluation, 7 out of 51 patients (14%) who disclosed interpersonal trauma were recommended a relevant further support organisation.

## Discussion

This audit provides an overview of how the IAT is being operationalised in CMHTs in North-East Glasgow to assist with routine sensitive enquiry of adult interpersonal trauma. The audit benefitted from a relatively large audit sample size of 90, this ensured a robust within sample size of 51 to answer the additional audit aims. In addition, by stratifying the sample, results were not over-representative of one service or discipline, but instead equal numbers of patients were reviewed from each CMHT. Therefore, the final sample reflected a variety of clinicians completing the initial assessment.

The findings of this audit provide a baseline of information to better understand how clinicians gather routine sensitive enquiry using the IAT. This **is** in keeping with previous ascertains of the benefits of assessment frameworks to identify victims of abuse (Agar et al., 2002). Most new assessments cited experiences of adult interpersonal trauma, which is indicative of its prevalence as a risk factor in the development and maintenance of mental health disorders (Okuda et al., 2011). This finding is suggestive of a role for services in supporting CMHT staff with the impact of completing trauma-focussed clinical assessment routinely, due to risks associated with compassion fatigue and burnout (Towey-Swift & Whittington, 2021). Therefore, using a framework such as this may have the additional benefit in assisting clinicians with role clarity during assessment phase with patients, which has been highlighted as a protective factor from burnout in CMHT staff (O’Connor et al., 2018).

Psychiatric diagnoses did not appear to influence the likelihood of enquiry into adult interpersonal trauma. This finding is in contrast with previous literature which suggests that patients with diagnoses, such as psychosis, are less likely to be asked about a history of abuse (Read et al., 2016). This may indicate the benefits of a standardised protocol, such as the IAT, to prompt clinicians to enquiry. In contrast, male participants were significantly less likely to have documented evidence of enquiry into adult interpersonal trauma. This may be a result of questions related to adult interpersonal trauma in the IAT indicating specific supplementary questions to be asked to women. While this gender-sensitive model of care is endorsed within the literature (Kiss et al., 2020) the template may have led clinicians to miss opportunities to support disclosures from male patients. This may be important to consider as males are significantly less likely to seek help following interpersonal trauma, compared to women (Huntley et al., 2019). In addition, help seeking behaviours and mental health presentations may also differ across genders (Donovan & Barnes, 2020; Kiss et al., 2020).

There was a drop-off in the rate of disclosures of adult interpersonal trauma informing patient risk assessment. This may indicate a learning need for assessing clinicians of the risks of revictimization for those with interpersonal trauma histories (Butler et al., 2020). It may be particularly pertinent for patients presenting with mental health disorders, as they are at increased vulnerability to interpersonal harm (Christ et al., 2020). It should be noted, however; that collecting data for the purposes of risk assessment has been demonstrated to be of little assistance in informing the future safety of victims (Turner et al., 2019). Therefore, the rationale for this data collection, such as informing case management and treatment planning, should be communicated with clinicians.

Fifty casefiles were excluded from the sample as their record was either without an IAT, or only a BAT was recorded. While this audit would be unable to ascertain the reasons why an IAT was not completed, the high number of missing records would seem to indicate a learning need for clinicians in operationalising local policy arrangements. As the BAT excludes questions pertaining to experiences of interpersonal trauma, it may be useful to understand if there are barriers to clinicians enquiring about abuse histories (Young et al., 2001). In addition, it may be advantageous to understand if there has been limited training on how to ask about histories of abuse (Lotzin et al., 2019), as well as knowledge-sharing regarding its prevalence within mental health presentations (Mauritz et al., 2013). Indeed, trauma-related training has been demonstrated to have advantageous outcomes for both frequency of enquiry, as well as greater detection of trauma by mental health professionals, so there is rationale for encouraging learning across staff groups (Coyle et al., 2019; Read et al., 2018a).

Less than 1 in 6 patients disclosing adult interpersonal trauma had demonstrable evidence of relevant additional support services being offered in relation to their experiences. This may be a missed opportunity to assist patients, given the positive impact social supports can have for the mental health of victims of interpersonal trauma (Ogbe et al., 2020). It may be that the indicated treatment was offered solely by the CMHT, so clinicians did not document cases where signposting advice was turned down, or it was not deemed suitable to signpost to such organisations during an initial assessment. It may also indicate a gap in clinician knowledge of local service provisions for adult survivors of interpersonal trauma. Therefore, building communication, training pathways and connectedness between NHS-service providers and voluntary organisations may build clinician confidence in routine sensitive enquiry (McCausland et al., 2021). Indeed, recent research has suggested that secondary care providers would benefit from improved pathways and integration strategies with the voluntary sector for enhancing patient experience and sustaining positive outcomes (Dayson et al., 2020). It may be beneficial for future audits to consider which services are being signposted to, with a view to better informing staff of ways to integrate resources between NHS and voluntary organisations.

## Limitations

Findings from this audit may not capture an accurate picture of the frequency of routine sensitive enquiry of adult interpersonal trauma. For example, clinicians may have not deemed it necessary to document an absence of trauma history in the IAT template. In addition, patients without a completed IAT were excluded from the dataset. Future research would benefit from examining the casefiles of patients with an incomplete, or missing, IAT to understand factors contributing to the lack of documentation. It may be that reason for referral influenced likelihood of routine sensitive enquiry. For example, enquiry into interpersonal trauma experiences for those presenting with psychotic experiences, have been under-reported in the literature (Neill & Read, 2022). Similarly, the influence of gender, namely being a male patient or male clinician, has been demonstrated to decrease rate of disclosure and enquiry respectively within disclosures of childhood abuse (Read et al., 2018b).

The utility of terms related to adult interpersonal trauma may have limited the identification of trauma histories from patients. For example, one question pertains to the patient having experienced various forms of interpersonal trauma. This may miss cases as the individual may not recognise it as abusive or traumatic (Hine et al.,  2022).

Disclosures of abuse to healthcare professionals depend on the context with which questions are asked, patient readiness and relationship with the healthcare professional (Feder et al., 2006). Indeed, it is imperative that routine sensitive enquiry considers any potential harms that may result from routine sensitive enquiry (Gentry & Paterson, 2022). Qualitative analysis of patient experiences of disclosures, as well as qualitative analysis of barriers in clinicians’ experience of engaging in routine sensitive enquiry of adult interpersonal trauma, would be timely.

## Conclusion

Across the 90 new patients evaluated in this audit from three Northeast Glasgow CMHTs, 57% (n = 51) of patients had evidence of the questions relating to routine sensitive enquiry being asked and documented within the IAT. Of these 51 patients, 31 had evidence of the information gathered from their IAT informing the CRAFT risk assessment. In terms of routine sensitive enquiry leading to the offer of additional support beyond CMHT intervention, this was the case in 7 out of the 51 patients in this audit. This review concludes that while the IAT can demonstrate clinicians’ routine sensitive enquiry of adult interpersonal trauma in mental health settings, more understanding is required around the use of disclosure information, as well as barriers to enquiry, when assisting patients within CMHTs. It is recommended that its current utility be reviewed by Services and additional training be provided to enhance case management and treatment planning.

## Recommendations

1. Given the prevalence of interpersonal trauma experienced by patients presenting to CMHT, trauma-informed approaches to care should be adopted. This would seek to assist patients, as well as clinicians, in working with trauma survivors in mental health settings.
2. There may be advantage in promoting the utility of the IAT to assist in informing risk assessments and promoting engagement with relevant additional support services.
3. Clinicians should be encouraged to assist patients in understanding what is meant by the term ‘abuse’.
4. Clinicians should be assisted with the psychological impact of assessing trauma disclosures in such frequency; this may be through models of reflective practice, clinical supervision, access to additional psychological support and practical support such as shared workloads.
5. As this audit intended to provide a baseline, it is recommended that it be repeated across a broader range of services in NHS GG&C to gather insight into how routine sensitive enquiry is currently being promoted with the IAT across the health board.
6. Future audits may benefit from understanding barriers to routine sensitive enquiry within the IAT, with consideration for tailoring enquiry for specific CMHT populations and demographics.
7. A final recommendation is that service managers consider improving links with local third sector organisations in order to aid collaborative working and signposting procedures.

## Dissemination

Results from this audit can be used to support the three CMHTs that were evaluated, and the research team will invite any questions or reflections. The executive summary and final written report was circulated more widely across NHS GG&C to support staff learning. There was also the opportunity to use findings to assist the current Board-wide strategies such as the Domestic Abuse Strategic Action Plan which sought input and consultation from local clinicians.

### Supplementary Information

Below is the link to the electronic supplementary material.

Supplementary file1 (DOCX 440 KB)
