# Supplementary material for: Routine Sensitive Enquiry of Adult Interpersonal Trauma in Community Mental Health Teams: An Audit of the Initial Assessment Tool
Source: Community Ment Health J. 2024 Feb 16;60(4):691–8. doi: 10.1007/s10597-023-01220-z (PMC11001718; doi:10.1007/s10597-023-01220-z)
Supplement: Supplementary file 1 — Supplementary file1 (DOCX 440 KB) [file 10597_2023_1220_MOESM1_ESM.docx]

**Appendices**

**Appendix 1: Initial Assessment Tool General Guidance Document.**

**Initial Assessment Tool Guidance**

- To be used for new patient assessments in place of the SSA
- Risk assessment to be completed separately as per existing guidance
- To be used as a template to guide assessment rather than as a list of questions
- It is not exhaustive and use clinical judgement on areas to focus on if necessary
- A copy of completed assessment to be sent to referrer with a covering letter as communication

| CHI No: |  | | | | | | | | Date: | | |  | | | |  |
| --- | --- | --- | --- | --- | --- | --- | --- | --- | --- | --- | --- | --- | --- | --- | --- | --- |
| Surname: |  | | | | | | | | Date Of Birth: | | |  | | | |  |
| First Name: |  | | | | | | | | Gender: | | |  | | | |  |
| Address &  Post Code: |  | | | | | | | | | | | | | | |  |
| Phone No^:^ |  | | | | | | | | Legal status  (MHA, AWI, Guardianship): | | |  | | | |  |
| Ethnicity: |  | | | | | | | | Nationality: | | |  | | | |  |
| Next of kin/ Carer: |  | | | | | | | | Consent to sharing info: | | |  | | | |  |
| NoK/Carer  contact details: |  | | | | | | | | Communication needs: | | |  | | | |  |
| Alerts *(history of violence, offending history)* | | | | | | | | | | | | | | | |  |
| Review emis alerts, risk screens and past notes. Check CareFirst if appropriate | | | | | | | | | | | | | | | |  |
| Reason for referral*(referrer’s reason[s] for requesting assessment)* | | | | | | | | | | | | | | | |  |
| For example a GP may refer an individual with concerns relating to mood or unusual behaviours which they worry may be psychotic. It might be helpful to document the referral descriptor. | | | | | | | | | | | | | | | |  |
| Reason for attendance *(Description of individual’s main concerns, their perceptions of difficulties and hopes from the service. Includes systemic enquiry of aspects such as sleep and appetite as well as impact on functioning)* | | | | | | | | | | | | | | | |  |
| A statement, in their own words, as to why they are there. This section is a subjective account; what they say and feel.  Get a sense of what is happening, for how long, potential reasons or triggers/ stressors, what helps or makes worse, if they are concerned about the situation or if others may have expressed concerns.  Further exploration of groups of symptoms e.g. mood, anxiety, unusual beliefs, psychotic symptoms, alcohol or illicit drugs as relevant to presenting complaint. | | | | | | | | | | | | | | | |  |
| Psychiatric history*(previous/ongoing mental health problems, diagnoses and interventions, via GP or mental health services, and their impact; h/o self-harm/attempted suicide; previous admission /detentions)* | | | | | | | | | | | | | | | |  |
| Remember this may need to be explored in a way the individual relates to, e.g. They may not have sought advice but may give an account of previous periods of ill health.  What has been helpful in the past? Take detail of past attempts or thoughts of harm. | | | | | | | | | | | | | | | |  |
| Medical history *(prior/pre-existing/ ongoing physical health problems, diagnoses and interventions and their impact)* | | | | | | | | | | | | | | | |  |
| Clinical portal may be a useful resource in this regard. | | | | | | | | | | | | | | | |  |
| Current medication details as given by individual*(prescribed, over-the-counter, complementary, drug allergies)* | | | | | | | | | | | | | | | |  |
| Consider if they are taking what is prescribed, if there is possible use of other non-prescribed meds  (E.g. from another person or bought from the internet.) Consider safe care of medications e.g. do they have immediate access/ stockpiling or excess medications at home. Independent managing medication or support provided and by whom. | | | | | | | | | | | | | | | |  |
| Medication | | | | | | Dose | | Frequency | | | Duration | | | | Response |  |
|  | | | | | |  | |  | | |  | | | |  |  |
|  | | | | | |  | |  | | |  | | | |  |  |
|  | | | | | |  | |  | | |  | | | |  |  |
|  | | | | | |  | |  | | |  | | | |  |  |
|  | | | | | |  | |  | | |  | | | |  |  |
|  | | | | | |  | |  | | |  | | | |  |  |
|  | | | | | |  | |  | | |  | | | |  |  |
|  | | | | | |  | |  | | |  | | | |  |  |
|  | | | | | |  | |  | | |  | | | |  |  |
|  | | | | | |  | |  | | |  | | | |  |  |
|  | | | | | |  | |  | | |  | | | |  |  |
|  | | | | | |  | |  | | |  | | | |  |  |
| Allergies: | | | | |  | | | | | | | | | | |  |
| Dispensing frequency: | | | | | E.g. Daily, weekly, dossette box | | | Medication concordance: | | | | |  | | |  |
|  | | | | | | | | | | | | | | | |  |
| Family History*(relationships; psychiatric, medical, substance use problems; h/o suicide)* | | | | | | | | | | | | | | | |  |
| What supports are available- this may not be family of origin.  Remember that the individual may be adopted or have grown up in a care setting.  Ask about suicide of others who may be close to the individual. | | | | | | | | | | | | | | | |  |
| Personal History: | | | | | | | | | | | | | | | |  |
| *Childhood/ development/ relationships*  *Childhood trauma/ neglect/ abuse*  *Education/ employment*  *Adult relationships*  *Adult trauma/ abuse/ vulnerability*  *Personal strengths/ Hopes for the future* | | | Use headings as a guide rather than an exhaustive list of questions. Be aware these can offer detail on potential neurodevelopmental issues (e.g. at birth), quality of home environment and early life, traits, relationship quality, early modelling of experiences/reactions, trauma, strength/resilience.  History of birth trauma, place of birth, family circumstances in childhood  Early (preschool) childhood; developmental difficulties, temperament  Parent-child relationship, attachments, siblings, making of friendships  Routinely enquire about childhood sexual abuse in addition to other forms of abuse and neglect as part of the initial assessment in a sensitive and supportive manner  Educational achievement and experience at school, conduct issues.  Employment history; professional relationships, why posts ended, further education  Relationship history; romantic and platonic  Routine Sensitive Enquiry  History of physical, emotional, or sexual abuse. History of financial exploitation  (Questions about Gender based violence for women)   - Have you ever experience physical, psychological or sexual abuse or violence within any of your intimate relationships? - Does the violence you’ve experienced still affect your well being, health or life? - Is there any physical, psychological or sexual violence or abuse in your current intimate relationships?   Religious beliefs, resilience | | | | | | | | | | | | |  |
|  | | | | | | | | | | | | | | | |  |
| Current Social Circumstances:  Use headings provided. Can consider exploration along the following lines: | | | | | | | | | | | | | | | |  |
| *Housing*  *Employment/*  *Employability*  *Finances*  *Support*  *Interests/*  *current activities* | | | Type, if secure tenancy, any concerns  If in employment or training, are they absent at present, work//vocational related stressors e.g. disputes/interpersonal difficulties, is work satisfying? Employability Pathway e.g. Where on pathway the individual is, aspirations.  Consider if referral to Occupational Therapy is indicated for vocational rehabilitation/job retention  Income and any concerns. Details of benefits if relevant  Available resources e.g. Family, friends, services (GP/counsellor/voluntary organisations)  Meaningful activities, how they spend their day, sense of satisfaction with this, are they achieving what they would wish to? | | | | | | | | | | | | |  |
| Any children/ dependents under the age of 18? | | | | | | | | | | | | | | | | |
| May need to cross check information in event of concern (e.g. Social Work Information System)  Consider other children who might be in household or contact e.g. non-biological, grandchildren | | | | | | | | | | | | | | | | |
|  | | | | | Child 1 | | | Child 2 | | | Child 3 | | | | Child 4 | |
| Name | | | | |  | | |  | | |  | | | |  | |
| Age | | | | |  | | |  | | |  | | | |  | |
| Address if different: | | | | |  | | |  | | |  | | | |  | |
| Child protection concerns/ agencies involved: | | | | |  | | |  | | |  | | | |  | |
| Impact of mental health on parenting and/or potential risk to children: | | | | | | | | | | | | | | | | |
| Awareness of direct and indirect harm/risk. Impact of illness, substances, age inappropriate roles in household (e.g. a child taking on carer responsibilities)  May need to consider intervention by appropriate agencies e.g. carer support, social work, health visitor. | | | | | | | | | | | | | | | | |
| Pre morbid personality: | | | | | | | | | | | | | | | | |
| Note- individuals may not give an accurate account of own personality. Collateral information can be invaluable.  Areas to explore include: what the individual was like prior to this episode/illness, how would their friends describe them, quality of friendships- superficial, intense, if easily formed and maintained, prevailing mood, interests, responses to stressors and difficulties. | | | | | | | | | | | | | | | | |
|  | | | | | | | | | | | | | | | | |
| Substance use *(caffeine, alcohol, tobacco, illicit, novel psychoactive substances)* | | | | | | | | | | | | | | | | |
| *Pattern of current use*  *Impact on individual*  *Previous history* | | | Consider if use in a dependent fashion or intermittent. Method of use (e.g. injecting and associated risks).  Impact on life e.g. employment, forensic, relationships, financial, health  Any abstinent periods, treatments and success of these. | | | | | | | | | | | | | |
|  | | | | | | | | | | | | | | | | |
| Forensic history *(Charges, fines, convictions, h/o violence)* | | | | | | | | | | | | | | | | |
| Past and pending charges, previous convictions, imprisonment, history of aggression and violence (even if not charged). | | | | | | | | | | | | | | | | |

|  | | | |
| --- | --- | --- | --- |
| Legal issues *(driving, capacity, detention, vulnerability, other orders)* | | | |
| Consider if subject to mental health or other legislation e.g. CTO, ASP, conditions of bail/court treatment orders.  Driving- if a license holder and if driving. Should they be advised not to drive (e.g. due to poor concentration, ideas of suicide re car, substance use, delusional beliefs re car). DVLA/police involvement may be necessary in some cases. Consider if immediate need to act or if it can be discussed e.g. at MDT. | | | |
|  | | | |
| Individuals expectation of service | | | |
| Record in their own words.  Hopes from attendance and understanding of what next steps/timeframes will be. | | | |
| Mental State Examination  Observations and comments may include the following: | | | |
| *Appearance*  *Behaviour*  *Mood & Affect*  *Speech*  *Thought form*  *Thought content*  *Perceptions*  *Cognition*  *Insight* | Dress and self-presentation, abnormal movements (tics, agitation, retardation of movement)  Rapport and engagement, if clearly responding to unseen stimuli, disinhibition, agitation  Give a subjective and objective account i.e. What the individual says their mood is and what is observed. Examples might include anxious, high or elated, low, fearful, numb. What is displayed on the face of the individual- are they labile, incongruous, flattened, effusive.  Is it possible to follow what is said? Is the speech normal in rate, tone and volume or does the individual display pressure or poverty of speech, flight of ideas. Is there thought disorder e.g. does the train of thought jump or suddenly stop, does the individual persist with a particular response despite the conversation moving on.  Preoccupations, concerns, delusional beliefs.  Can include abnormal beliefs e.g. overvalued ideas. Delusions and abnormal experiences e.g. hallucinations (of any modality), passivity experiences, depersonalization or derealisation.  Can include orientation, attention and concentration, memory, language, fluency, visuospatial skills (certain screens may be used e.g. Mini mental state examination, Addenbrookes).  Does the individual identify or consider there is a problem which they wish to address? Do they feel treatment or help is required? What is this, in their opinion? Do they have a realistic awareness of risks/concerns? | | |
|  | | | |
| Collateral history *(including carers/next of kin/others expectations from service)* | | | |
| Explore their account of events and concerns. Potential triggers  What they feel would be helpful and what outcome they expect from the assessment.  Consider if this individual warrants a carers assessment or other supports. | | | |
|  | | | |
| Additional notes | | | |
| This may include a wide variety of information or considerations.  E.g. need to consider historical CSA allegations, involvement of NOK with certain exclusions re areas permitted to share, particular personal requests with regard care and treatment. | | | |
|  | | | |
| Summary of assessment *(Key findings, relevant negatives, initial formulation)* | | | |
| Brief summary of significant clinical information, significant findings at history and mental state. Statement re risks. Consider predisposing, precipitating, perpetuating, factors. | | | |
| Immediate actions *( including information provided)* | | | |
| Document any immediate treatment decisions, information provided tothe individual or brief interventions  These may include- immediate actions re risks (to self and others e.g. thoughts of suicide, harm to others, driving), medication needs (e.g. short course of sedative or hypnotic, removal of meds), physical health care needs (e.g. if acute infection, delirium felt central).  There should always be a senior member of staff available to discuss any immediate concerns.  Update Alerts on EMIS is relevant | | | |
|  | | | |
| Outcome of MDT discussion/ Treatment plan *(including follow up arrangements)* | | | |
| Document MDT view of assessment outcome and decision on future care provision. Include details of any follow up arrangements, medication changes and future input form the services. It would important to include information about other services, resources the individual may be sign posted to as an outcome of the assessment or in the future. It might be helpful to consider making recommendations for future management or resources that the referrer may be able to access if the individual is not offered a further service. | | | |
| Primary Diagnosis: |  | | |
| Any additional diagnoses: |  | | |
| Suitable for psychological therapies: Y / N | | | |
| Name: |  | Designation: |  |
| Signature |  | Date: |  |

**Appendix 2: Adult Mental Health Initial Shared Assessment (IAT) Principles and Guidelines for Completion Document.**

**Adult Mental Health Initial Shared Assessment (IAT) Principles and Guidelines for Completion**

This guidance note is not intended to be exhaustive in nature but provide general principles for practice and use of the documentation.

# Introduction & General Principles

The underlying ethos and approach to assessment is to be person-centred, strengths based, recovery and outcome focused. It should allow the exploration and examination of the individual’s difficulties as well as strengths.

The Initial Assessment Tool (IAT) simply provides a standardised format upon which information can be recorded. This aids the recording and sharing of information.

The order of the questions should not drive the way in which the assessment is undertaken. The information gathered at assessment should then be recorded within the patient’s electronic record.

The assessment can be enhanced with additional information from a variety of information sources Including families and carers where appropriate.

The IAT replaces the specialist shared assessment and related assessment tools currently within use across GG&C. The IAT will be used for all new assessments within Adult CMHTs, Crisis and home treatment services and adult acute inpatient services within GG&C Mental Health Services.

The IAT and supplementary information does not substitute the need for good multi‐disciplinary integrated working, communication and service user involvement but should enhance our ability to document and evidence it.

*What are the key reasons for having a Mental Health Initial Assessment?*

- 1. A core assessment which meets the need for recording of relevant details to support sharing of information.
  2. A standardised assessment recording tool used across the GG&C Adult Mental Health Services.
  3. To collect information to support multidisciplinary decision making

# Operational guidance

The IAT has two versions, a full and a brief version. All new routine referrals will have a full IAT completed as part of the assessment process. The brief IAT can be used in the following scenarios:

- Urgent and emergency assessment
- Crisis and IHTT initial assessment
- If a full assessment has been completed within the previous 6 months

Where a briefer assessment tool has been used, the full IAT version will be completed within 28 days of initial contact for all patients who continue to receive input from the service at that stage. The Clinical Risk Assessment Framework Tool (CRAFT) should be completed at the point of assessment and at appropriate intervals as indicated within the Clinical Risk Screen Policy. Additional risk assessment documents may also be used where agreed by the service.

The tool is designed to be used by all professional groups undertaking initial assessments within the previously mentioned settings. Where an alternative format, such as a letter, is used to communicate with the referrer, it is the responsibility of the assessor to ensure that it covers all relevant sections within the tool.

The tool is designed to be a dynamic document and, depending on the individual’s difficulties, service setting and other circumstances, might require to be completed over more than one contact. It is important that the tool is updated with the outcome of any MDT discussions that have been undertaken and the agreed treatment plan. All record keeping will be as per existing organizational and professional record keeping standards. The IAT does not replace the requirements placed on individual members of staff or the multi‐disciplinary team to ensure there is clear communication and dialogue as per existing standards.

# Governance, audit and assurance processes

The local Service/Operations Manager will have responsibility for the governance and assurance of local standards across services. The document template within EMIS is the only version that will be used to ensure version control.

**Supplementary 1: Approval form from Caldicott Guardian.**

**
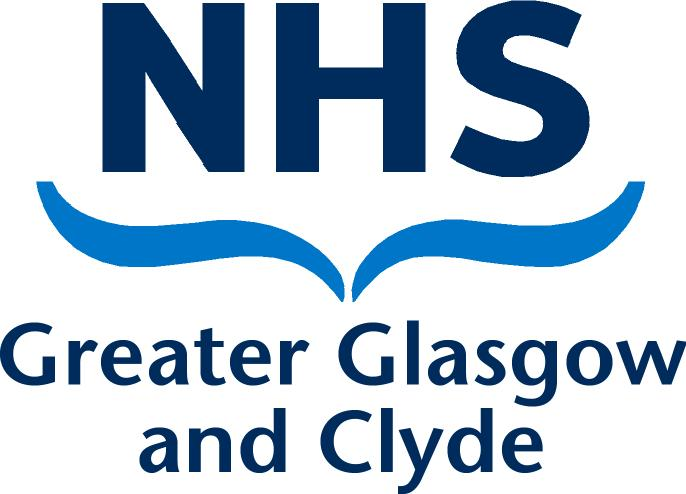
** **Application for Caldicott Guardian Approval**

**NOTE: You must address the 6 Caldicott principles (Appendix A) when submitting this application.**

**1. Study/project title**

|  |
| --- |

1. **Please tick the type of study/project you are undertaking**

| Audit ☑ Research □ Service Improvement □ Other □  If other, please provide further details: |
| --- |

1. **Who is providing clinical support for the study/project**

(NB this should be someone from NHSGGC such as a Clinical Director and be different from the person requesting the information).

| Name: ANONYMISED FOR BLIND REVIEW  Designation: Clinical Psychologist  Email Address or Telephone Number: |
| --- |

**4. Details of individual/organisation requesting data**

| Name: ANONYMISED FOR BLIND REVIEW  Designation: Trainee Clinical Psychologist  Work/University Address: Gartnavel Royal Hospital, 1055 Great Western Rd, Glasgow, G12 0XH  Contact Details: |
| --- |

**5. Purpose for which data are to be used** (Principle 1)

| The data will be used to explore how the Initial Assessment Tool is being used across 3 CMHT’s in North East Glasgow. Specifically, questions relating to routine sensitive enquiry of adult trauma will be considered. |
| --- |

**6. Which identifiable data items are required? Please detail why these are required.** (Principles 2 and 3)

| **PID Required** |  | **Justification** |
| --- | --- | --- |
| CHI Number |  | EMIS number will be used to identify patient in the first instance and will correlate to participant ID.  I.e; participant 1 = EMIS 12345678 |
| Forename |  |  |
| Surname |  |  |
| DOB |  |  |
| Age |  |  |
| Gender |  |  |
| Address |  |  |
| Post code (full) |  |  |
| Post code (partial) |  |  |
| Clinical data |  |  |
| Other (please specify) |  |  |

**7. Who will have access to this information?** (Principle 4)

| Internal: ANONYMISED FOR BLIND REVIEW  External: Nobody |
| --- |

**8. Storage and use of personal data during the audit/project** (Principle 5)

| Will you be undertaking any of the following activities at any stage (including the identification of potential participants)? Please tick all which are appropriate.  □ Access to health record (paper)  □X Access to health record (electronic)  □ Sharing of identifiable data with other organisations (provide further detail below)  □ X Publication of data (if this could identify individuals provide further detail below)  *Data will be published as part of a Doctorate in Clinical Psychology qualification and will also be disseminated within the service.* ***No identifiable data will be published.***  □ Use of audio/visual recording devices  Storage of personal identifiable data on any of the following:  □ Manual files, including x-rays  □ X NHS computers  □ Home or other personal computers  □ University computer  □ Private company computer  □ Laptop computer (or any other mobile device)  □ USB flash drive  *The data will be cleaned before transferring it onto a USB flash drive.* |
| --- |
| Additional Information:  **Publication of data will be non-identifiable information only**. |

**9. Destruction of Data**

| How long will the data be held?  The anonymised data will be held on a secure NHS laptop for the duration of the audit.  How will the data be destroyed?  The data will be destroyed by deleting the spreadsheets with anonymised information on stored on the NHS laptop. |
| --- |

**10. Please provide your organisation’s Data Protection Registration Number**

(if external to NHSGGC)

|  |
| --- |

**Note:**

- Copies of any other relevant supporting documentation (e.g. ethics approval, patient information leaflet etc.) should be attached to this application.
- Appendix A details the Caldicott Principles.

**Person responsible for the requested data:**

Name**: ANONYMISED FOR BLIND REVIEW**

Designation: Trainee Clinical Psychologist

Signature **ANONYMISED FOR BLIND REVIEW** Date 29/04/2022...........................

The release of data as described above is: Approved

**Caldicott Guardian …**
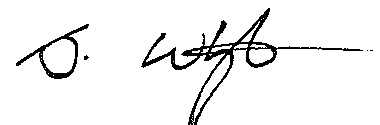
**Date …05/05/2022….**
